# Extending the Martini 3 Coarse-Grained Force Field to Carbohydrates

**DOI:** 10.1021/acs.jctc.2c00553

**Published:** 2022-07-29

**Authors:** Valery Lutsyk, Pawel Wolski, Wojciech Plazinski

**Affiliations:** † Jerzy Haber Institute of Catalysis and Surface Chemistry, Polish Academy of Sciences, Niezapominajek 8, 30-239 Krakow, Poland; ‡ Department of Biopharmacy, Medical University of Lublin, Chodzki 4a, 20-093 Lublin, Poland

## Abstract

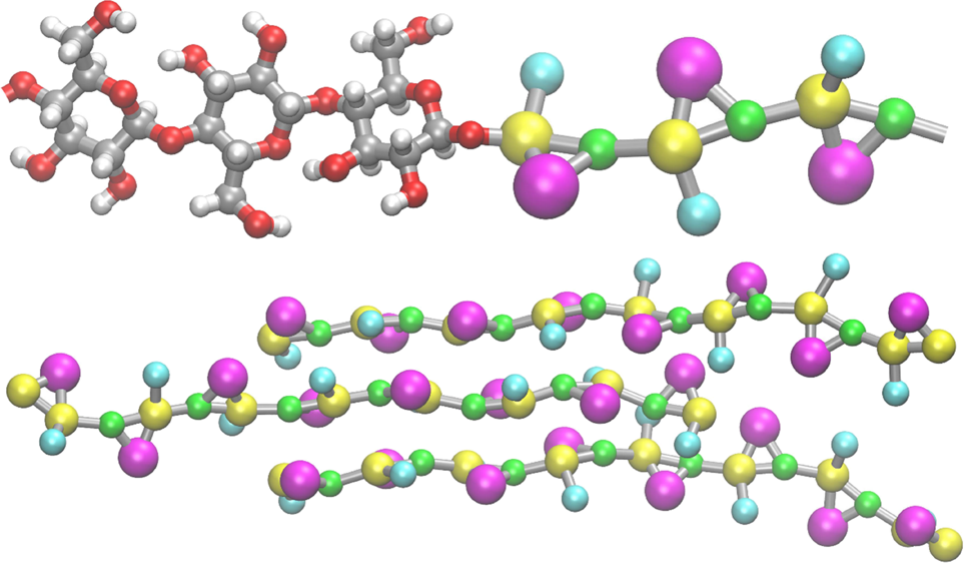

Carbohydrates play an essential role in a large number of chemical and biochemical processes. High structural diversity and conformational heterogeneity make it problematic to link their measurable properties to molecular features. Molecular dynamics simulations carried out at the level of classical force fields are routinely applied to study the complex processes occurring in carbohydrate-containing systems, while the usefulness of such simulations relies on the accuracy of the underlying theoretical model. In this article, we present the coarse-grained force field dedicated to glucopyranose-based carbohydrates and compatible with the recent version of the Martini force field (v. 3.0). The parameterization was based on optimizing bonded and nonbonded parameters with a reference to the all-atom simulation results and the experimental data. Application of the newly developed coarse-grained carbohydrate model to oligosaccharides curdlan and cellulose displays spontaneous formation of aggregates of experimentally identified features. In contact with other biomolecules, the model is capable of recovering the protective effect of glucose monosaccharides on a lipid bilayer and correctly identifying the binding pockets in carbohydrate-binding proteins. The features of the newly proposed model make it an excellent candidate for further extensions, aimed at modeling more complex, functionalized, and biologically relevant carbohydrates.

## 1 Introduction

Carbohydrates (saccharides) play an essential and widely recognized role in numerous chemical,^1,2^ biochemical,^3,4^ and technological processes^5,6^ as well as display potential for the design of new materials.^7−9^ A series of carbohydrate properties (chemical heterogeneity, variable glycosidic linkage types, variable functionalization patterns, nonuniform chain length, and high conformational heterogeneity even at the monomer level) make studying them at the molecular level problematic.^10,11^ As an alternative to experimental approaches, the theoretical methods of molecular simulations can be proposed in order to quantitatively investigate the carbohydrate-containing systems.

Molecular dynamics (MD) simulations are a commonly applied computational tool capable of providing the connection between the molecular-level structure of the given system and its measurable properties. The usefulness of the MD simulations relies primarily on the accuracy of the underlying potentials of interactions assigned to describe the system (force fields). Numerous atomistic (all-atom, AA) and united-atom force fields have been parametrized for either unfunctionalized or functionalized carbohydrates and have been used to provide conformational, structural, and thermodynamic details related to saccharide properties.^12,13^ Inherent to MD simulations, the system size often becomes a bottleneck for simulation efficiency. Thus, simulations of long polysaccharide chains or large, carbohydrate-containing systems may be very challenging when considering the atomistic level of resolution.

A convenient alternative to the AA-based simulations is the use of coarse-grained (CG) force fields.^14−17^ Such choice significantly enhances the computational efficiency associated with simulations because of both reducing the system size and increasing the timestep parameter. As a consequence, large systems can be studied on a microsecond-long timescale and at reasonable computational cost. There exist several carbohydrate-dedicated CG models.^18−22^ However, in most of the cases, a serious limitation is that the given model is restricted to the sole class of carbohydrates and there is no compatible set of models capable of describing other types of molecules and biomolecules that often coexist with carbohydrates in real systems. In 2009, the Martini-based CG model for unfunctionalized, glucopyranose-based carbohydrates has been proposed.^23^ This model, its subsequent extensions^24−26^ and modifications^27^ are compatible with the earlier version of the Martini 2 force field, enabling simulations of complex systems, containing, for example, lipid bilayers and disaccharides,^28^ covalently bonded glycans,^29^ crystalline carbohydrates,^26^ and many other carbohydrate-containing systems.^30,31^ At the same time, several shortcomings of this model have been reported,^32^ including, for example, overestimated self-aggregation properties.^27^ Most of these shortcomings resulted from the underlying inaccuracies of the nonbonded interactions in the Martini 2-inherent, Lennard-Jones (LJ) parameters for certain bead types combined with the center-of-mass approach used for bead mapping. Recently, an updated version of the Martini force field has been developed,^33^ differing substantially from the previous one and improving most of the spurious effects. However, the presently distributed force field parameters lack more complex carbohydrates and are limited to only two monosaccharide units (glucose and ribose). It is also worth noting that possible directions of extending the Martini 3.0 model toward the carbohydrates have been explored in a previous study;^34^ however, none of the solutions proposed there has been included in the Martini distribution so far.

**Figure 1:**
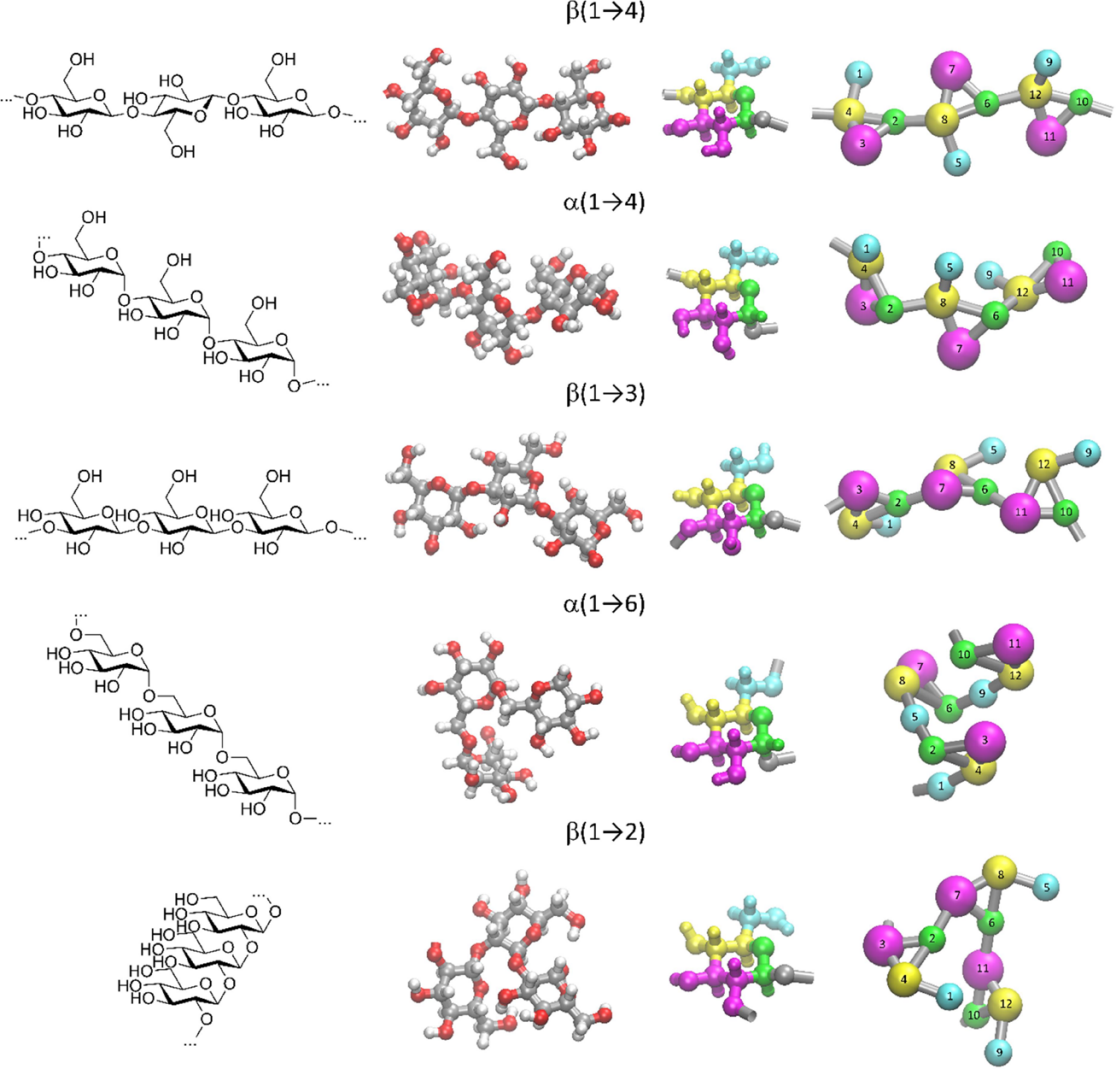
Chemical formulas of the considered glucose-based saccharides exploiting different linkage types, atomistic structures of the corresponding trimers, and the illustration of the mapping scheme on the example of a single residue. The shown bead numbering is used to define the force field terms (Tables 3–7). Analogous bead numbering is applied in the case of monosaccharides (Table 2).

In this work, we present the extension of the recently developed Martini 3.0 CG force field, including the parameters for glucopyranose-based carbohydrates. The parameterized group comprised the carbohydrate di-, oligo-, and polysaccharides exploiting the most important glycosidic linkages (i.e., β(1 → 2), β(1 → 3), α(1 → 4), β(1 → 4), and α(1 → 6)). The set of compounds considered by us is given in Figure 1. The procedure of parameterization relied on the use of atomistic data (MD simulations carried out at an AA level of accuracy) and experimental measurements (log *P* values, structural data). The final parameter set was tested by carrying out a series of CG MD simulations of different, glucose-based saccharides in water as well as in the presence of other biomolecules (lipid bilayers and proteins).

The article is organized as follows: The Theoretical Grounds section contains the accepted mapping procedure, the brief description of the force field functional form and the force field build-up rules. The Methods section includes the detailed description of the computational methodology used throughout the study and concerning either AA or CG simulations. The Model section, divided into several subsections with respect to considered aspects, reports the final CG parameters. The section entitled Properties of Studied Systems includes a series of simulation results that provide a validation for the newly proposed force field with respect to various carbohydrate-containing systems (including the comparison with the available experimental data and AA simulations). The discussion on the force field applicability and its possible limitations is given in the subsequent section. Finally, Conclusions summarize the main findings and provide concluding remarks.

## 2 Theoretical Grounds

### 2.1 General Remarks

The proposed CG model concerns the glucose-containing saccharides, in particular, glucopyranose monomers (as either α or β anomers), disaccharides containing the linkages of the β(1 → 2), β(1 → 3), α(1 → 4), β(1 → 4), or α(1 → 6) types, as well as oligo- and polysaccharides exploiting such linkages. The model relies on the nonbonded parameters introduced for the Martini 3.0 force field,^33^ which is developed by using the Martini-inherent strategies of parameterization, designed to be compatible with the Martini water model and was tested in combination with existing Martini models, describing other types of systems (e.g., lipid bilayers and proteins). Therefore, it can be considered as compatible with the Martini 3.0 family of CG force fields. In spite of that, the presently proposed approach substantially differs from the prospective carbohydrate-dedicated models discussed in the previous study.^34^ In the following subsection, we provide a brief overview of the basic parametrization methodology, including procedures followed for the definition of the mapping scheme and parametrization of nonbonded and bonded interactions.

### 2.2 Mapping

According to the mapping scheme accepted in Martini 3.0, the three different CG beads can be used to represent two (tiny bead, T), three (S, small bead), or four (R, regular bead) heavy atoms. Therefore, at least three CG particles are required to model a single glucose monosaccharide. In contrast to the previous edition of the Martini carbohydrate-dedicated model, compatible with Martini 2,^23^ we decided to use uniform, four-bead representation for a single glucose residue either as monosaccharide or being a part of a longer, oligo/polysaccharide chain. According to the proposed mapping, the carbohydrate residue is divided into four beads, representing the following molecular regions: (1) the hydroxymethyl group; (2) the ring oxygen, anomeric carbon and (in the case of reducing end or monosaccharide) anomeric hydroxyl group; (3) the vicinal diol or hydroxyethyl group (depending on the linkage type); and (4) the hydroxyethyl group. The graphical illustration of the accepted mapping scheme is given in Figure 1.

Although such scheme increases the number of beads per one unfunctionalized hexopyranose residue from 3 to 4 and, subsequently, slightly decreases the computational efficiency inherent to the model, it also has several advantages concerning both the present work and prospective model extensions:

1. Increasing the resolution of the model that represents the polysaccharide backbone which is now composed of either two ((1 → 2), (1 → 3), (1 → 4) linkages), or three ((1 → 6) linkages) bonds per residue in a chain. This increases the accuracy of the model with respect to structural rearrangements along the carbohydrate main chain. In particular, the rotation around the (1 → 6) linkages can now be properly modeled, which was impossible in the previous version of the carbohydrate-dedicated Martini 2 model.^23^
2. Reflecting the structural and topological features of carbohydrate polymers. This includes the symmetry of the carbohydrate residue and its basic geometrical features. For instance, the central part of the glucose ring is composed of the two nonbonded groups of atoms; therefore, it is represented by two separate beads.
3. Uniform mapping for either monosaccharides or residues in a chain. This feature facilitates perspective polysaccharide-related model extensions and its refinements based on the single-residue models.
4. Facilitating the procedure of prospective parameterization for functionalized polymers composed of pyranose residues. In the case of adding some ring-substituents, only minor refinements in the parameters concerning the closest neighbors of those substituents will be required. The most essential part of the model, that is, that describing the properties of glycosidic bonds, will remain nearly unaffected. This is analogous to the parameterization procedures developed for carbohydrates in some of the atomistic force fields.^35,36^

The mapping relied on the center-of-geometry (COG) approach introduced for Martini 3.0.^33^ The COG approach includes all atoms contributing to a given CG bead, together with aliphatic hydrogens. In order to account for this, the AA MD simulations, performed according to the methodology described below, were selected as the source of the target data, concerning mainly the bonded CG parameters.

### 2.3 Bonded Interactions

The functional form of the potential-energy term, associated with the stretching of CG bonds and applied to all unique pairs of covalently linked beads, is given by eq 1:
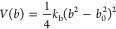
1where *b* is the bond-length distance, *b*_0_ is its reference value, and *k*_b_ is the corresponding force constant.

The functional form of the potential-energy term, associated with the bending of bond angles and applied to selected triplets of covalently linked beads, is given by eq 2:
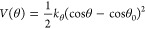
2where θ is the bond-angle value, θ_0_ is its reference value, and *k*_θ_ is the corresponding force constant. In certain cases, especially when the bond angle becomes close to 180°, the special type of bond-angle bending potential (restricted bending potential)^37^ is used in order to prevent the numerical errors and simulation instabilities:
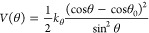
3where all variables are defined as in eq 2.

The functional form of the potential-energy term, associated with the deformation of improper-dihedral angles and applied to the subset of bead quadruplets in order to control out-of-plane distortions, is given by eq 4:
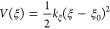
4where ξ is the improper-dihedral angle value, ξ_0_ is its reference value, and *k*_ξ_ is the corresponding force constant.

Finally, the functional form of the potential-energy term, associated with the torsion around dihedral angles, is given by eq 5:
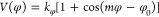
5where φ is the dihedral angle value, *m* is the multiplicity of the term, φ_0_ is the associated phase shift, and *k*_ϕ_ is the corresponding force constant. This term is applied to a subset of all possible dihedral angles as specified in Section 4 and Tables 3^4^,5,6,7.

Functions defined by eqs 1–5 are invoked by using the following types of interaction functions in GROMACS: eq 1: bonds, type 1; eq 2: angles, type 2; eq 3: angles, type 10; eq 4: dihedrals, type 2; eq 5: dihedrals, type 1.

### 2.4 Nonbonded Interactions

The considered nonbonded interactions are represented solely by LJ potentials because of electrical neutrality of all considered compounds and their building blocks. The nonbonded interactions are calculated as a sum over all interacting nonbonded pairs (*i*,*j*) using the following 12/6 interaction function with parameters C_12_ and C_6_:
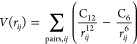
6where *r_ij_* is the distance between interacting beads. The parameters C_12,*ij*_ and C_6,*ij*_ depend on the type of involved beads and those used in the present work were chosen among the bead types derived for Martini 3. Following the Martini convention, the first covalent neighbors are excluded from nonbonded interactions.

During selection of the optimal LJ parameters, we considered the chemical character of the mapped functional groups, the AA-derived solvent-accessible surface area (SASA) values, the experimental log *P* values measured for glucose monosaccharides, and the theoretically (KOWWIN and ALOGPS) predicted log *P* for selected di- and oligosaccharides.

## 3 Methods

### 3.1 CG Simulations

The detailed list of the systems studied at the CG level is given in Table 1. The initial structures were either drawn manually or generated by using the hand-written *python3* program *carbo2martini3.py* (included in the Supporting Information). The *insane* tool was used to solvate the considered solute molecule and construct the initial configuration of the lipid bilayer wherever needed. The parameters concerning saccharides and constituting the currently proposed force field are given and discussed in detail in further sections. The remaining types of molecules (lipids, proteins) were modeled by using the Martini 3.0 parameters^33^ and prepared by using the web-available tools (*martinize2* and *insane*). Simulations were carried out with the GROMACS 2016.4 package,^38^ under periodic boundary conditions and in the isothermal–isobaric ensemble. The temperature was maintained close to the reference value (298, if not indicated otherwise) by applying the V-rescale thermostat,^39^ whereas for the constant pressure (1 bar), the Parrinello-Rahman barostat^40^ was used with a relaxation time of 40 ps. Either semi-isotropic (bilayer systems) or isotropic (remaining systems) pressure scaling was applied. The equations of motion were integrated with a time step of 10 (β(1 → 3)-glucan- and β(1 → 2)-glucan-containing systems), 30 (monosaccharides), or 20 fs (remaining systems) using the leap-frog scheme.^41^ The translational center-of-mass motion was removed every timestep separately for the solute and the solvent. The Martini 3 water model^33^ was applied. The van der Waals interactions (LJ potentials) are shifted to zero beyond the cutoff distance, that is, 1.1 nm. For Coulomb interactions, the reaction-field approach was used with a cutoff of 1.1 nm and ε_r_ = 15. Details of the MD parameters were kept according to the sample *mdp* files deposited at the *cgmartini.nl* website. Production simulations were carried out for a duration of 100–10,000 ns (depending on the system), and the data were saved to trajectory every 10–50 ps.

**Table 1:** CG Systems Considered in the Present Study^a^

purpose	compositon	no. of solvent molecules	box vector lengths [nm^3^]	simulation time	remarks
conformational properties, SASA	α-Glc monomer	323	3.0 × 3.0 × 3.0 nm^3^	5000 ns	unbiased MD
conformational properties, SASA, log *P*	β-Glc monomer	323	3.0 × 3.0 × 3.0 nm^3^	5000 ns unbiased MD,20 ns per TI window	Unbiased MD, TI, various nonbonded parameters
log *P*	β-Glc monomer	450	4.8 × 4.8 × 4.8 nm^3^	20 ns per TI window	TI, octanol solvent, various nonbonded parameters
solution density	100–2000 β-Glc monomers	1514–14,177	12 × 12 × 12 nm^3^	10 ns	unbiased MD, various Glc content
lipid bilayer	DPPC lipid bilayer	11,645–11,663	10 × 10 × 18 nm^3^	500 ns	unbiased MD,various temperatures
influence on the lipid bilayer	4200 β-Glc monomers + DPPC lipid bilayer	4242–4299	10 × 10 × 23.5 nm^3^	500 ns	unbiased MD, various temperatures
log *P*	α(1 → 4)-linked Glc octamer	8100	9.9 × 9.9 × 9.9 nm^3^	50 ns per TI window	TI, various nonbonded parameters
log *P*	α(1 → 4)-linked Glc octamer	5064	10.8 × 10.8 × 10.8 nm^3^	50 ns per TI window	TI, octanol solvent, various nonbonded parameters
conformational properties, SASA	β(1 → 4)-linked Glc dimer	3354	7 × 7 × 7 nm^3^	500 ns	unbiased MD
conformational properties, SASA	α(1 → 4)-linked Glc dimer	3353	7 × 7 × 7 nm^3^	500 ns	unbiased MD
conformational properties, SASA	β(1 → 2)-linked Glc dimer	3348	7 × 7 × 7 nm^3^	500 ns	unbiased MD
conformational properties, SASA	β(1 → 3)-linked Glc dimer	3350	7 × 7 × 7 nm^3^	500 ns	unbiased MD
conformational properties, SASA	α(1 → 6)-linked Glc dimer	3351	7 × 7 × 7 nm^3^	500 ns	unbiased MD
conformational properties, SASA	β(1 → 4)-linked Glc tetramer	4871	8 × 8 × 8 nm^3^	500 ns	unbiased MD
conformational properties, SASA	α(1 → 4)-linked Glc tetramer	4872	8 × 8 × 8 nm^3^	500 ns	unbiased MD
conformational properties, SASA	β(1 → 2)-linked Glc tetramer	4870	8 × 8 × 8 nm^3^	500 ns	unbiased MD
conformational properties, SASA	β(1 → 3)-linked Glc tetramer	4874	8 × 8 × 8 nm^3^	500 ns	unbiased MD
conformational properties, SASA	α(1 → 6)-linked Glc tetramer	4874	8 × 8 × 8 nm^3^	500 ns	unbiased MD
conformational properties, SASA	β(1 → 4)-linked Glc octamer	9187	10 × 10 × 10 nm^3^	500 ns	unbiased MD
conformational properties, SASA	α(1 → 4)-linked Glc octamer	9195	10 × 10 × 10 nm^3^	500 ns	unbiased MD
conformational properties, SASA	β(1 → 2)-linked Glc octamer	9181	10 × 10 × 10 nm^3^	500 ns	unbiased MD
conformational properties, SASA	β(1 → 3)-linked Glc octamer	9187	10 × 10 × 10 nm^3^	500 ns	unbiased MD
conformational properties, SASA	α(1 → 6)-linked Glc octamer	9197	10 × 10 × 10 nm^3^	500 ns	unbiased MD
aggregation properties	3 β(1 → 3)-linked Glc chains (26 residues)	29,364	15 × 15 × 15 nm^3^	200 ns	unbiased MD, initiated from triple-helix structure
aggregation properties	1 α(1 → 4)-linked Glc chain (32 residues)	132,476	25 × 25 × 25 nm^3^	250 ns	unbiased MD, initiated from V-helix structure, various solvents: water and 1-bead solvents (C1 to C6)
aggregation properties	50 α(1 → 4)-linked Glc octamers	271,906	32 × 32 × 32 nm^3^	1500 ns	unbiased MD
aggregation properties	100 β(1 → 4)-linked Glc octamers	112,182	24 × 24 × 24 nm^3^	4575 ns	unbiased MD
aggregation properties	10 β(1 → 3)-linked Glc octamers	35,376	16 × 16 × 16 nm^3^	10,000 ns	unbiased MD
aggregation properties	50 α(1 → 4)-linked Glc polymers (26 residues)	606,152	42 × 42 × 42 nm^3^	170 ns	unbiased MD
aggregation properties	50 β(1 → 4)-linked Glc polymers (26 residues)	606,248	42 × 42 × 42 nm^3^	780 ns	unbiased MD
aggregation properties	40 β(1 → 3)-linked Glc polymers (26 residues)	124,888	25 × 25 × 25 nm^3^	650 ns	unbiased MD
interactions with proteins	1 protein (PDB: 1I8A) and + 1 monomer of β-Glc	8579 + 98 Na^+^ + 91 Cl^–^	10 × 10 × 10 nm^3^	10,000 ns	unbiased MD
Interactions with proteins	1 protein (PDB: 4O7P) and + 25 maltose molecules	27,444 + 310 Na^+^ + 294 Cl^–^	15 × 15 × 15 nm^3^	5000 ns	unbiased MD
Interactions with proteins	1 protein (PDB: 2OVW) and + 25 cellobiose molecules	27,750 + 305 Na^+^ + 307 Cl^–^	15 × 15 × 15 nm^3^	10,000 ns	unbiased MD

a If not indicated otherwise, the solvent was Martini 3 water, for which 1 CG bead = 4 water molecules.

The log *P* values were calculated as the Gibbs free energy difference corresponding to the transfer of the saccharide molecule from water to *n*-octanol. The calculations concerned the following systems: monosaccharide of β-d-glucopyranose and α(1 → 4)-linked octamer of glucopyranose. In order to construct the thermodynamic cycle, the carbohydrate molecule was decoupled (removed) from both water and *n*-octanol solvents. Decoupling the carbohydrate molecule from either type of system was carried out by scaling down to zero all nonbonded interactions involving carbohydrate atoms in a stepwise manner as a function of a coupling parameter λ. The associated free energy changes were calculated with the Bennett acceptance ratio (BAR) method,^42^ implemented in the GROMACS *gmx bar* subroutine, including the error estimation determined by using the default criteria. The 21 evenly spaced λ-points were accepted, and the data from equilibrated systems were collected every 1 ps for a duration of 100 (monomers) or 500 ns (octamers) in each λ window. A soft-core function was used for the van der Waals interactions to prevent energy singularities.

### 3.2 AA Simulations

The following saccharides were considered during AA simulations: (1) monosachcarides of α- and β-d-glucopyranose; (2) α(1 → 4)-linked octamers of glucopyranose; (3) β(1 → 4)-linked octamers of glucopyranose; (4) β(1 → 3)-linked octamers of glucopyranose; (5) β(1 → 2)-linked octamers of glucopyranose; (6) α(1 → 6)-linked octamers of glucopyranose; (7) disaccharides linked by each of the above linkage types. Additionally, the data from our previous carbohydrate-oriented studies were occasionally used to validate the CG model. The CHARMM36^43,44^ force field was used in all AA simulations. The initial structures of saccharides as well as the GROMACS-readable parameters were generated by the www.charmm-gui.org online server.^45,46^ Simulations were carried out with the GROMACS 2016.4 package.^38^ The saccharide molecules were placed in simulation boxes of dimensions dependent on the system type and surrounded by a number of explicit water molecules approximately accounting for the system density of 1 g/cm^3^. The MD simulations were carried out under periodic boundary conditions and in the isothermal–isobaric ensemble. The temperature was maintained close to its reference value (298 K) by applying the V-rescale thermostat,^39^ whereas for the constant pressure (1 bar, isotropic coordinate scaling), the Parrinello–Rahman barostat^40^ was used with a relaxation time of 0.4 ps. The equations of motion were integrated with a time step of 2 fs using the leap-frog scheme.^41^ The translational center-of-mass motion was removed every timestep separately for the solute and the solvent. The TIP3P model of explicit water^47^ was applied and the full rigidity of the water molecules was enforced by the application of the SETTLE procedure.^48^ The hydrogen-containing solute bond lengths were constrained by the application of the LINCS procedure with a relative geometric tolerance of 10^–4^.^49^ The electrostatic interactions were modeled by using the particle-mesh Ewald method^50^ with cutoff set to 1.2 nm, while van der Waals interactions (LJ potentials) were switched off between 1.0 and 1.2 nm. Production simulations were carried out for a duration of 100 ns, and the data were saved to trajectory every 2 ps.

## 4 Model

### 4.1 Parameters

#### 4.1.1 Nonbonded Interactions

**Figure 2:**
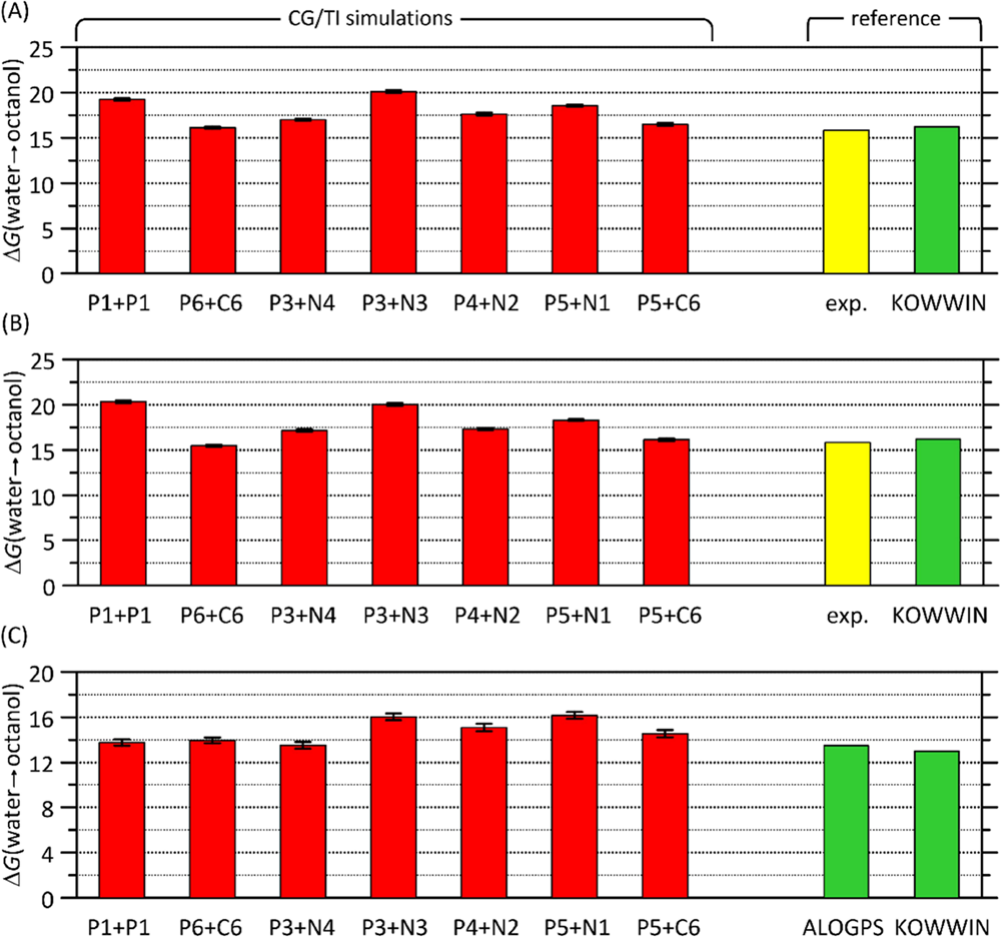
Results of the CG TI simulations aimed at determining the free energies associated with the transfer of glucose mono- and oligosaccharides from water to *n*-octanol (equivalent to log *P*). (A) Results for monosaccharides with rings composed of T, S, and R beads. (B) Results for monosaccharides with rings composed of T, S, and S beads. (C) Results for octamers with rings composed of T, S, and R beads (recalculated with respect to a single residue). Theoretical data are compared with predictions of KOWWIN (Estimation Programs Interface Suite for Microsoft Windows, v 4.11) and ALOGPS 2.1^51^ programs as well as with the experimental data.^52^

**Figure 3:**
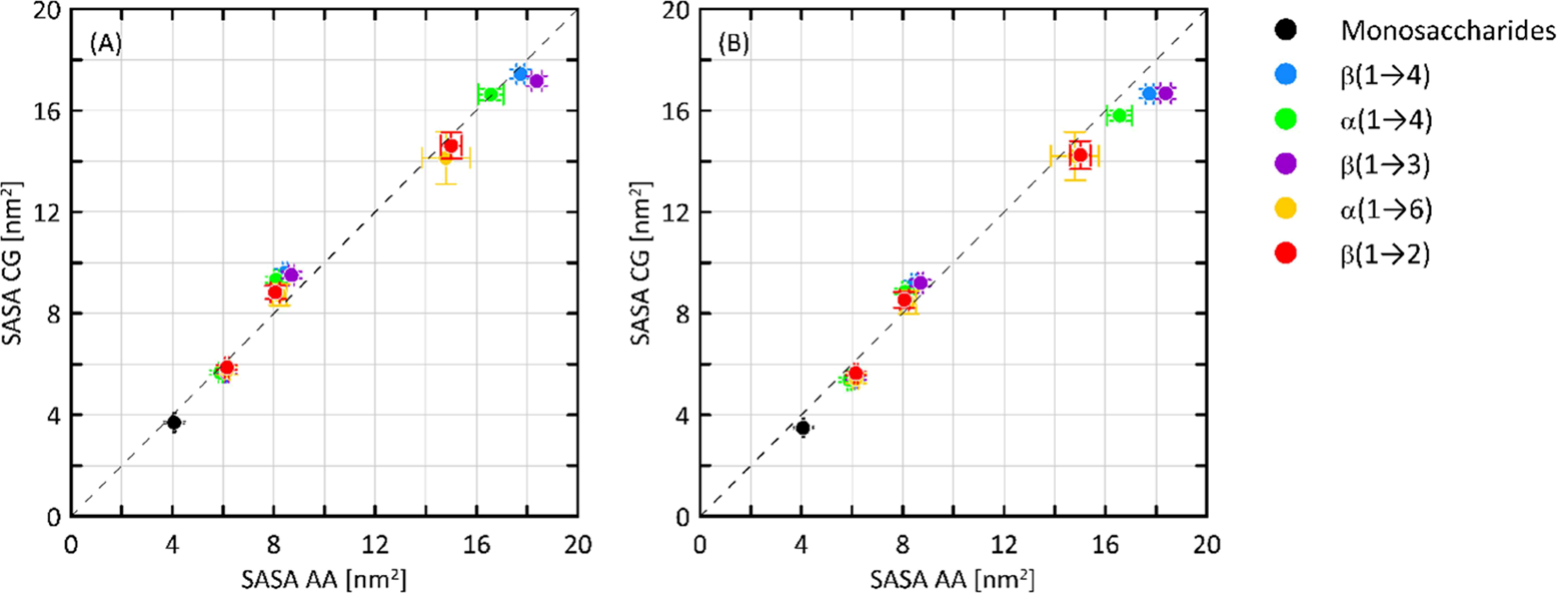
Comparison of the SASA values obtained from either AA of CG simulations. Two alternative assignments of the bead type for one of the ring beads were accepted: (A) S bead; (B) R bead.

The chemical character of glucopyranose monomer partially dictates the assignment of nonbonded parameters that should take into account the following factors: (1) the similar or the same polarity of beads representing the hydroxymethyl and diol groups (beads of odd numbers; see Figure 1); (2) the similar or the same polarity of beads representing the central part of the monosaccharide or polysaccharide backbone (beads of even numbers; see Figure 1); (3) more polar character of odd beads in comparison to less polar even beads. Multiple combinations fulfilling these conditions have been tested. The results for selected combinations which provided the best agreement with the reference data are illustrated in Figure 2.

A very similar level of agreement with the experimental data was achieved in each of the cases shown in Figure 2. Moreover, the trend of decreasing polarity of the glucose oligomer in comparison to the monomer was correctly reflected. In view of these similarities, we decided to accept the TP3 and P3 bead types to describe the more polar, outward regions of the Glc molecule, whereas the inner part, belonging to the polymer backbone in the case of the Glc-based chains, was represented by the SN4 and TN4 beads. The choice of the P3 bead is in agreement with the polar character of hydroxymethyl and diol groups and with the 3-bead model for the glucose monomer existing in the current implementation of Martini 3.0. Its complementation by the N4 bead type is dictated by the match of the total polarity of the Glc molecule with the reference data.

Finally, it is worth noting that the change of one of the ring bead types from R to S has only marginal influence on the calculated log *P* values. The same can be stated in a more general context, about nearly all combinations of bead types, shown in Figure 2. This is discussed in further sections.

#### 4.1.2 Bonded Interactions

Table 2 contains the optimized bonded parameters proposed for α- and β-glucopyranose monomers. Because of structural similarities of both anomers, only some minor differences concerning the B2 bead are involved. Consequently, the parameters for remaining d- or l-aldohexopyranoses can straightforwardly be obtained by using only a limited set of alterations in the currently proposed parameters in accordance to the corresponding AA simulations.

**Table 2:** Parameters for Glucopyranose Monomers (Bond Stretching, Bond-Angle Bending, and Improper-Dihedral Distortion) in the Presently Proposed Force Field^a^

type	topological pattern	parameters			
		α-anomer		β-anomer	
		***k*_b_ [kJ mol^–1^ nm^–4^]**	***b*_0_ [nm]**	***k*_b_ [kJ mol^–1^ nm^–4^]**	***b*_0_ [nm]**
bonds:	B1-B4	12,000	0.268	12,000	0.268
	B2-B3	20,000	0.284	24,000	0.291
	B3-B4	28,000	0.291	28,000	0.291
	B2-B4	28,000	0.342	32,000	0.355
		***k*_θ_ [kJ mol^–1^]**	**θ_0_ [deg]**	***k*_θ_ [kJ mol^–1^]**	**θ_0_ [deg]**
angles	B1-B4-B2^b^	340	85	450	81
	B1-B4-B3^b^	580	132	580	132
		***k*_ξ_ [kJ mol^–1^ deg^–2^]**	**ξ_0_ [deg]**	***k*_ξ_ [kJ mol^–1^ deg^–2^]**	**ξ_0_ [deg]**
improper dihedrals	B4-B3-B2-B1	200	9	200	9

a Force constants correspond to eqs 1–4.

b Eq 2.

Parameters for saccharides containing two or more glucose units were developed separately for each of the following linkage types: β(1 → 2), β(1 → 3), α(1 → 4), β(1 → 4), and α(1 → 6). They are collected in Tables 3^4^,5,6,7. As mentioned, the accepted strategy of parameterization allows for exploiting the monosaccharide parameters in constructing the CG models for more complex saccharides. Thus, developing parameters for saccharide chains relied on adopting the monosaccharide-derived parameters (Table 2) in a nearly unchanged form and adding components responsible for modeling the glycosidic bonds. Thus, apart from relatively minor alterations resulting from reassignments of atoms contributing to beads connecting two adjacent residues, the monosaccharide parameters are fairly well conserved during this stage of parameterization.

**Table 3:** Parameters for α(1 → 4)-Linked Glucopyranose Di-, Oligo-, and Polysaccharides (Bond Stretching, Bond-Angle Bending, and Improper- and Regular-Dihedral Distortion) in the Presently Proposed Force Field^a^

type	topological pattern	parameters		
		***k*_b_ [kJ mol^–1^ nm^–4^]**	***b*_0_ [nm]**	
bonds:	B1-B4	18,000	0.251	
	B2-B3	32,000	0.280	
	B3-B4	34,000	0.279	
	B2-B4	60,000	0.292	
	B2-B8	32,000	0.280	
		***k*_θ_ [kJ mol^–1^]**	**θ_0_ [deg]**	
angles	B1-B4-B2^b^	340	86	
	B1-B4-B3^b^	580	142	
	B3-B2-B8^b^	310	103	
	B4-B2-B8^c^	180	103	
	B1-B2-B8^b^	210	105	
	B2-B8-B5^b^	30	107	
	B2-B8-B6^c^	240	148	
	B2-B8-B7^b^	120	99	
		***k*_ξ_ [kJ mol^–1^ deg^–2^]**	**ξ_0_ [deg]**	
improper dihedrals	B4-B3-B2-B1^d^	200	9	
	B8-B2-B7-B6	170	22.3	
	B2-B8-B4-B3	300	–67	
		***k*_ϕ_ [kJ mol^–1^]**	**ϕ_0_ [deg]**	***m***
regular dihedrals	B4-B2-B8-B6	–12.8	161	1
	B3-B2-B8-B7	–2.2	165	1
	B1-B3-B7-B5	–3.6	–72	5

a Force constants correspond to eqs 1–5. Atom numbering concerns only the first linkage; the parameters for any subsequent, *n*th linkage can be obtained by increasing the corresponding bead numbers by 4*n*.

b Eq 2.

c Eq 3.

d Concerns only the nonreducing end.

**Table 4:** Parameters for β(1 → 4)-Linked Glucopyranose Di-, Oligo-, and Polysaccharides (Bond Stretching, Bond-Angle Bending, and Improper- and Regular-Dihedral Distortion) in the Presently Proposed Force Field^a^

type	topological pattern	parameters		
		***k*_b_ [kJ mol^–1^ nm^–4^]**	***b*_0_ [nm]**	
bonds:	B1-B4	14,100	0.250	
	B2-B3	37,500	0.268	
	B3-B4	27,000	0.273	
	B2-B4	53,200	0.257	
	B2-B8	7500	0.267	
	B2-B6	16,300	0.520	
	B4-B8	3770	0.542	
		***k*_θ_ [kJ mol^–1^]**	**θ_0_ [deg]**	
angles	B1-B4-B2^b^	220	91	
	B1-B4-B3^c^	159	143	
	B3-B2-B8^b^	245	115	
	B1-B2-B8^b^	350	127	
	B2-B8-B5^b^	16	123	
	B2-B8-B7^b^	52	93	
		***k*_ξ_ [kJ mol^–1^ deg^–2^]**	**ξ_0_ [deg]**	
improper dihedrals	B4-B3-B2-B1^d^	200	9	
	B8-B2-B7-B6	212	11	
	B2-B3-B8-B4	229	9	
		***k*_ϕ_ [kJ mol^–1^]**	**ϕ_0_ [deg]**	***m***
regular dihedrals	B3-B2-B8-B7	–35	–135	1

a Force constants correspond to eqs 1–5. Atom numbering concerns only the first linkage; the parameters for subsequent, *n*th linkage can be obtained by increasing the corresponding bead numbers by 4*n*.

b Eq 2.

c Eq 3.

d Concerns only the nonreducing end.

**Table 5:** Parameters for β(1 → 3)-Linked Glucopyranose Di-, Oligo-, and Polysaccharides (Bond Stretching, Bond-Angle Bending, and Improper- and Regular-Dihedral Distortion) in the Presently Proposed Force Field^a^

type	topological pattern	parameters		
		***k*_b_ [kJ mol^–1^ nm^–4^]**	***b*_0_ [nm]**	
bonds:	B1-B4	10,000	0.268	
	B2-B3	38,000	0.240	
	B3-B4	24,000	0.287	
	B2-B4	50,000	0.287	
	B2-B7	12,000	0.250	
		***k*_θ_ [kJ mol^–1^]**	**θ_0_ [deg]**	
angles	B1-B4-B2^b^	220	78	
	B1-B4-B3^b^	400	123	
	B3-B2-B7^b^	60	75	
	B1-B2-B7^c^	80	148	
	B2-B7-B6^b^	110	128	
	B2-B7-B8^b^	40	65	
		***k*_ξ_ [kJ mol^–1^ deg^–2^]**	**ξ_0_ [deg]**	
improper dihedrals	B4-B3-B2-B1	120	15	
	B7-B2-B8-B6	200	22	
	B2-B7-B3-B4	200	3	
		***k*_ϕ_ [kJ mol^–1^]**	**ϕ_0_ [deg]**	***m***
regular dihedrals	B3-B2-B7-B6	–20	–174	1

a Force constants correspond to eqs 1–5. Atom numbering concerns only the first linkage; the parameters for subsequent, *n*th linkage can be obtained by increasing the corresponding bead numbers by 4*n*.

b Eq 2.

c Eq 3.

**Table 6:** Parameters for β(1 → 2)-Linked Glucopyranose Di-, Oligo-, and Polysaccharides (Bond Stretching, Bond-Angle Bending, and Improper-Dihedral Distortion) in the Presently Proposed Force Field^a^

type	topological pattern	parameters		
		***k*_b_ [kJ mol^–1^ nm^–4^]**	***b*_0_ [nm]**	
bonds:	B1-B4	10,000	0.265	
	B2-B3	50,000	0.263	
	B3-B4	24,000	0.272	
	B2-B4	50,000	0.290	
	B2-B7	6000	0.274	
	B4-B7	600	0.530	
		***k*_θ_ [kJ mol^–1^]**	**θ_0_ [deg]**	
angles	B1-B4-B2^b^	200	80	
	B1-B4-B3^b^	400	132	
	B3-B2-B7^b^	30	88	
	B1-B2-B7^c^	60	165	
	B2-B7-B6^b^	50	97	
	B2-B7-B8^c^	40	148	
		***k*_ξ_ [kJ mol^–1^ deg^–2^]**	**ξ_0_ [deg]**	
improper dihedrals	B4-B3-B2-B1	150	11	
	B7-B6-B8-B2	300	15	
	B2-B4-B3-B7	200	1	
		***k*_ϕ_ [kJ mol^–1^]**	**ϕ_0_ [deg]**	***m***
regular dihedrals	B3-B2-B7-B6	–22	–141	1

a Force constants correspond to eqs 1–5. Atom numbering concerns only the first linkage; the parameters for subsequent, *n*th linkage can be obtained by increasing the corresponding bead numbers by 4*n*.

b Eq 2.

c Eq 3.

**Table 7:** Parameters for α(1 → 6)-Linked Glucopyranose Di-, Oligo-, and Polysaccharides (Bond Stretching, Bond-Angle Bending, and Improper- and Regular-Dihedral Distortion) in the Presently Proposed Force Field^a^

type	topological pattern	parameters		
		***k*_b_ [kJ mol^–1^ nm^–4^]**	***b*_0_ [nm]**	
bonds:	B1-B4	21,000	0.251	
	B2-B3	35,000	0.280	
	B2-B4	60,000	0.322	
	B3-B4	24,000	0.289	
	B2-B5	13,000	0.225	
	B3-B7	2400	0.820	
	B1-B9	180	0.790	
		***k*_θ_ [kJ mol^–1^]**	**θ_0_ [deg]**	
angles	B1-B4-B2^b^	600	80	
	B1-B4-B3^b^	560	135	
	B3-B2-B5^b^	180	109	
	B4-B2-B5^b^	100	105	
	B2-B5-B6^c^	28	122	
		***k*_ξ_ [kJ mol^–1^ deg^–2^]**	**ξ_0_ [deg]**	
improper dihedrals	B4-B2-B3-B1	250	–7	
	B2-B3-B5-B4	110	–74	
		***k*_ϕ_ [kJ mol^–1^]**	**ϕ_0_ [deg]**	***m***
regular dihedrals	B3-B2-B5-B6	–3	155	2
	B2-B5-B6-B7	–3	170	5
	B2-B5-B6-B7	3	60	1
	B2-B4-B8-B6	5	–25	1
	B2-B4-B8-B6	–4	165	3

a Force constants correspond to eqs 1–5. Atom numbering concerns only the first linkage; the parameters for subsequent, *n*th linkage can be obtained by increasing the corresponding bead numbers by 4*n*.

b Eq 2.

c Eq 3.

The conformation of CG saccharide chains can be described by the following two determinants: (1) the geometry of the backbone, defined, depending on the linkage type, by the following beads: 4–2–8–6–12–10–... (α(1 → 4) and β(1 → 4) linkages); 3–2–7–6–11–10–... (β(1 → 2) and β(1 → 3) linkages); and 1–4–2–5–8–6–9–12–10–... (α(1 → 6) linkage); (2) the orientation of the rings (represented by the triplets of atoms: 2–3–4, 6–7–8, ...) with respect to the backbone. In the case of non-α(1 → 6) linkages, the conformation of the backbone can be described by the two different types of quadruplets of atoms, creating repeating motifs: (1) quadruplet with two central atoms within the ring; (2) quadruplet with two central atoms creating the residue-residue linkage. The geometry of the dihedral angle included in the first of these motifs is controlled by the two improper-dihedral terms involving atoms that create linkages. Thus, because of the functional forms of the corresponding potential-energy terms (eq 4), the conformation of those backbone fragments will be represented by a single energy well. In contrast, the conformation of the dihedral angle associated with the second topological motif will be correlated with a rotation around the glycosidic linkage. However, the geometry of the same dihedral angle is convoluted in the rotation of neighboring rings around the backbone because of the restricted shapes of those rings. Thus, both abovementioned determinants are often treated collectively by introducing a series of improper-dihedral terms, controlling geometry within residue and adjacent, covalently bound beads and regular-dihedral term(s) controlling the rotations around glycosidic linkages. In the most complex case of the α(1 → 6) linkage, the additional regular-dihedral terms are required, combined with bonds between nonadjacent beads, in order to maintain proper conformation of a chain.

Let us note that in some cases, the bonds between nonadjacent beads were introduced in order to control the geometry of the triplets of atoms. This was done mainly in those systems where the geometry of those triplets becomes close to linear, resulting in potential numerical errors and simulation instability. For the same reasons, the regular bond angle term (eq 2) was replaced by the restricted-angle term (eq 3) for flexible bond angles with optimal values close to 180°.

### 4.2 General Remarks

The parameters collected in Tables 2–7 were developed, tested, and validated under assumption that the first covalently bound neighbors are excluded from any nonbonded interactions.

In most of the systems, the developed parameters allow to carry out the numerically stable MD simulations with a timestep of 0.02 ps or even 0.03 ps (monosaccharides). The exception are the systems containing either the β(1 → 2) or β(1 → 3) linkages where the probability of linearization of atoms triplets involved in either bond angle of the dihedral type of bonded interactions is too large. In the case of these two system types, the stability of MD simulations was achieved upon decreasing the timestep to 0.01 ps.

Interestingly, several features that seem to be largely dependent on the bead type (e.g., log *P*, SASA, density of glucose solutions; see details in further sections of the article) were relatively weakly affected by alternative assignments of the bead type. This includes both bead types and sizes (R, S, and T, according to the MARTINI-characteristic distinction). Moreover, the conformational properties of both monomers and octamers, relying mainly on the bonded parameters, are fairly independent of the choice of nonbonded parameters. Thus, although we have accepted the P3 + N4 combination as the final one, some other, chemically sound choices may also provide a reasonable modification of the model. The main motivation for introducing such alterations may be the modification of interaction strength either within the carbohydrate–carbohydrate pairs or between carbohydrates and other biomolecules or solvents.

## 5 Properties of the Studied Systems

The subsequent subsections report the results obtained by using the newly developed set of parameters to study various carbohydrate-containing systems. These results aim to provide a validation of the force field and, in part, were used to adjust the final parameters.

### 5.1 SASA

The SASA parameter values were calculated at both CG and AA levels of resolution for mono-, di-, and octasaccharides. For nonmonomeric saccharides, the calculations involved all considered glycosidic linkage types. The results are illustrated in Figure 3. This part of the study confirms that the CG model is capable of accurately reproducing the molecular surface and molecular volume properties, as essential for Martini 3.0-based models.^33,53^ Interestingly, replacing the R bead in the ring structures into the corresponding S one has a negligible influence on the calculated SASA values. In both cases, the relative and absolute deviations from the atomistic data are extremely small and of comparable magnitude.

### 5.2 Conformational Properties

The parameters collected in Tables 2–7 were used to produce a series of models for carbohydrate mono- and octamers, subsequently applied to validate the conformational properties against the predictions of AA simulations. This was done by comparing the distribution of selected conformational descriptors: (1) distances between selected CG bead pairs; (2) angles between triples of beads; (3) improper dihedrals defined by quadruplets of beads; (4) regular torsional angles defined by quadruplets of beads; (5) end-to-end distances; (6) gyration radii. Because of numerous possible combinations of beads defining a given descriptor, we limited ourselves to analyzing mainly those that can be directly traced back to certain terms in the force field (e.g., given bead-bead bond or dihedral angle defined on a rotatable bond). In the case of octamers, the analysis mentioned in points (1)–(4) always concerned central residue(s) of the chain. Covalent bead-bead bonds, bond angles, and dihedral terms are, as a rule, modeled by the harmonic potential (see the Model section). Thus, in order to simplify the comparison for descriptors of those types, we provide only the average values and the accompanying fluctuation measures (standard deviations on the set of values). Independent of that, the full comparison of the distributions was carried out during the stage of parametrization and data analysis. On the contrary, the regular-dihedral angles are reported as distributions recovered from AA and CG simulations.

**Figure 4:**
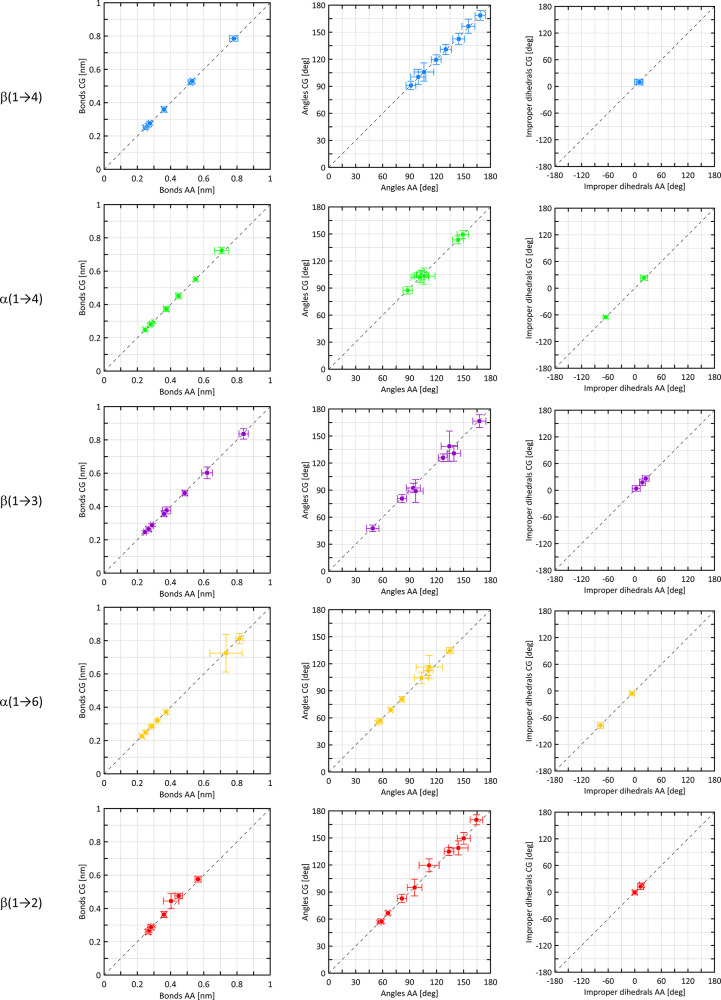
Comparison of the average values of the selected bead-bead distances, bead-bead-bead regular angles, and improper-dihedral angles calculated from unbiased MD simulations within either the AA or CG force field for different types of glycosidic linkages. Horizontal and vertical bars denote the fluctuation associated with a given descriptor, expressed as the standard deviation on the data set.

The results are given in Figure 4 (bonds, angles, and improper-dihedral average values), 5 (regular-dihedral distributions), and 6 (gyration radii and end-to-end value distributions). In most of the cases, the agreement between AA and CG data is excellent or at least satisfactory. In the case of bonds, bond angles, and improper dihedrals, most essential deviations are the consequence of the inherent asymmetry of the AA distributions approximated by symmetrical, parabolic potentials. However, deviations of this type are not frequent and the level of asymmetry as well as deviations from unimodal character of distributions are not large, which results in a reasonable level of agreement between AA and CG data (Figure 4). The most pronounced consequence in this context is ignoring the secondary conformers for polysaccharide backbone in chains composed of β(1 → 4) linkages. This approximation is quite accurate, as the population of neglected conformers is ca. 2.5% but can have some influence when studying backbone kinks in extremely long chains. At a smaller scale, it underestimates the rigidity of oligomeric chains containing β(1 → 4) linkages expressed by the end-to-end distance (deviation between average values equal to ca. 3.5%) but has very little influence on the gyration radii (deviation equal to ca. 1%). On the other hand, a series of independent CG simulations carried out for very long cellulose chains (>50 units) shows that the alternative measure of chain rigidity, that is, the persistence length, is overestimated in comparison to the available experimental data.

**Figure 5:**
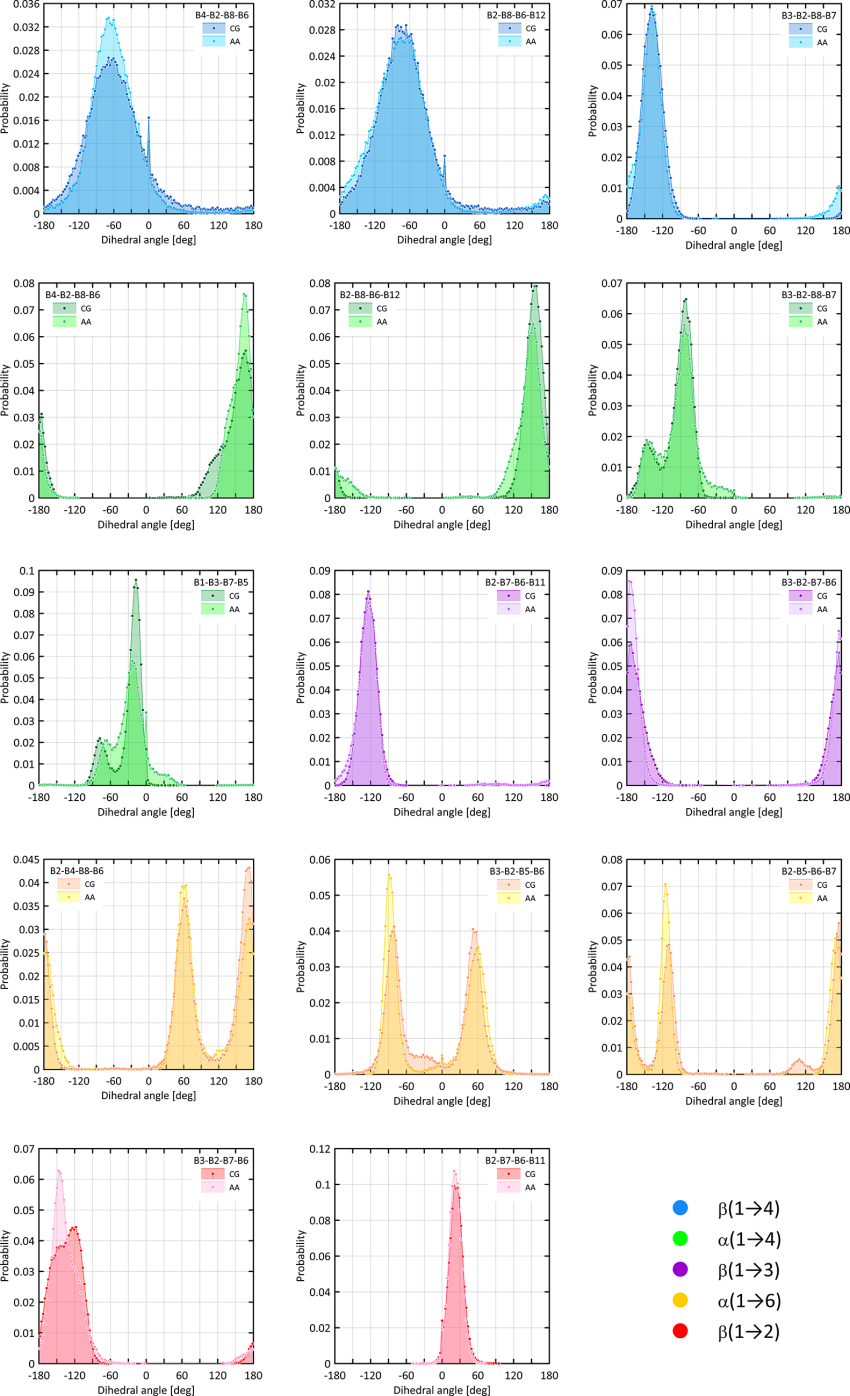
Distributions of dihedral angle values calculated within either the AA or CG force field. The angles correspond to the selected rotation around glycosidic linkages of different types.

**Figure 6:**
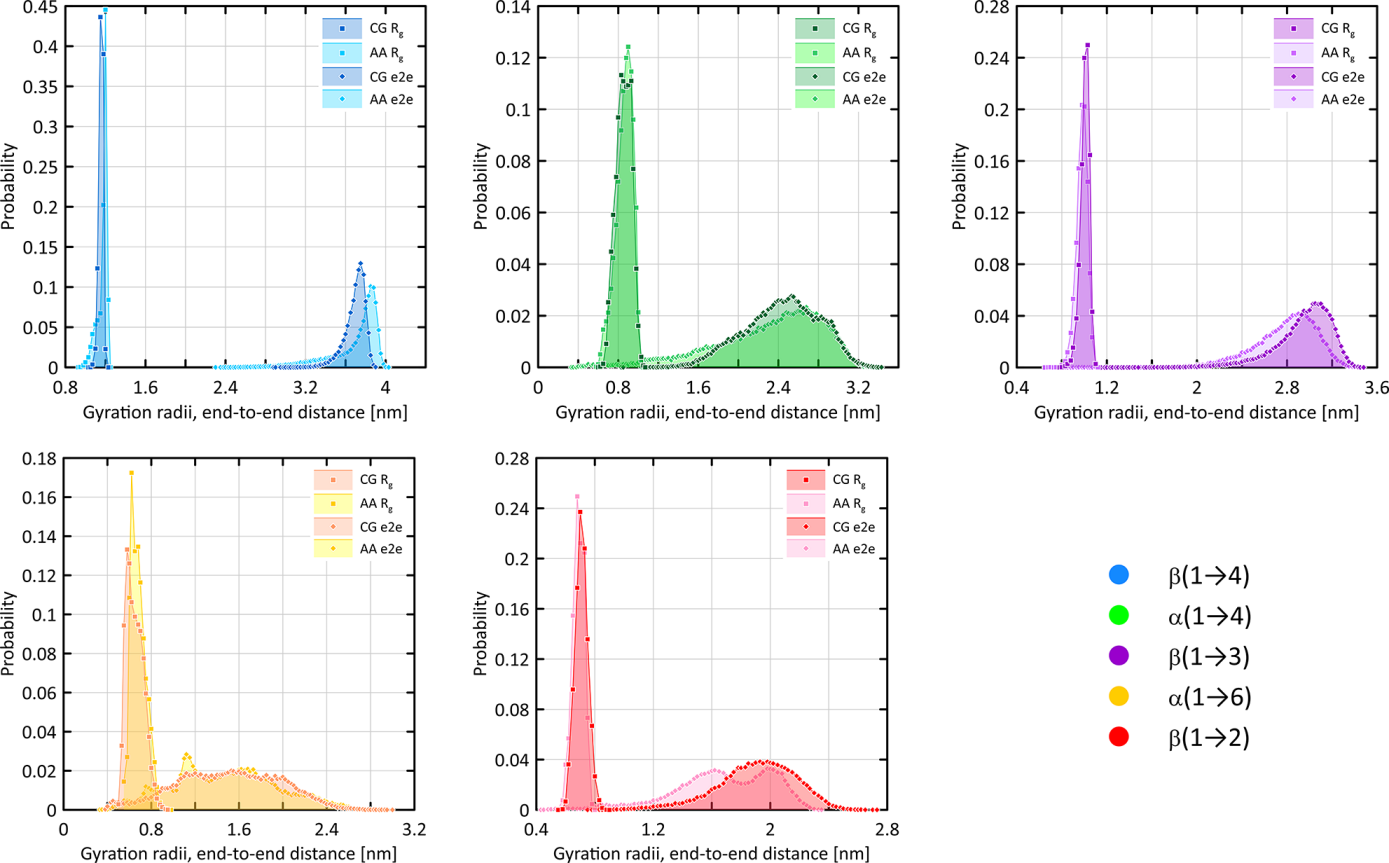
Distributions of gyration radii (*R*_g_) and end-to-end distance (e2e) values calculated within either the AA or CG force field. Calculations concerned homooctasaccharides containing different types of glycosidic linkages.

This effect and other potential inaccuracies concerning the case of long saccharide chains may result from the fact that the current CG model partially ignores the possibility of “kinks” along the carbohydrate backbone. Such kinks represent the secondary or tertiary conformers of glycosidic linkages (anti-ϕ and anti-ψ rotamers). However, the conformational flexibility within the main conformational state is reproduced very well, as shown in Figures 4−6. Moreover, the presence of anti-ϕ and anti-ψ rotamers in some of the glucose-based polysaccharides is partially accounted for by possible rotations of residues around the backbone. By allowing for that, we preserve the dynamic equilibrium between relative orientations of the B1 and B3 (or only B3 in the case of (1 → 6) linkages) beads of the neighboring residues even if the backbone shape does not reflect the correlated reorientation.

The accurate implementation of the “kinks” in the saccharide backbone will be the subject of our future studies, focused on those members of the carbohydrate family for which the flexibility of both backbone and rings plays a more significant role (e.g., the iduronate-containing glycosaminoglycans^11^).

The agreement between AA and CG data in the context of regular-dihedral angles is shown in Figure 5. Distributions obtained for linkages of the β(1 → 2), β(1 → 3), and β(1 → 4) types have a relatively simple character, indicating the existence of a single free energy well. In contrast, the distributions calculated for α(1 → 4) and α(1 → 6) linkages are more complex and associated with more than one well-defined conformational states. However, both these tendencies are satisfactory, reproduced by the CG model. The most significant deviations are often a consequence of inability to find the function modeling the CG torsion of multiplicity, which cannot be expressed by an integer (see eq 5).

Finally, the good agreement between polymer properties (gyration radii and end-to-end distance) calculated at CG and AA levels is worth mentioning (Figure 6). The larger deviations between average values are characteristic of the β(1 → 2), β(1 → 3), and β(1 → 4) linkages, exhibiting unimodal character of distributions of glycosidic torsional angle values (Figure 5). Nevertheless, in all of the cases, the average value and even the nonsymmetric character of AA distributions are reasonably well reflected by the corresponding CG model.

### 5.3 Density

**Figure 7:**
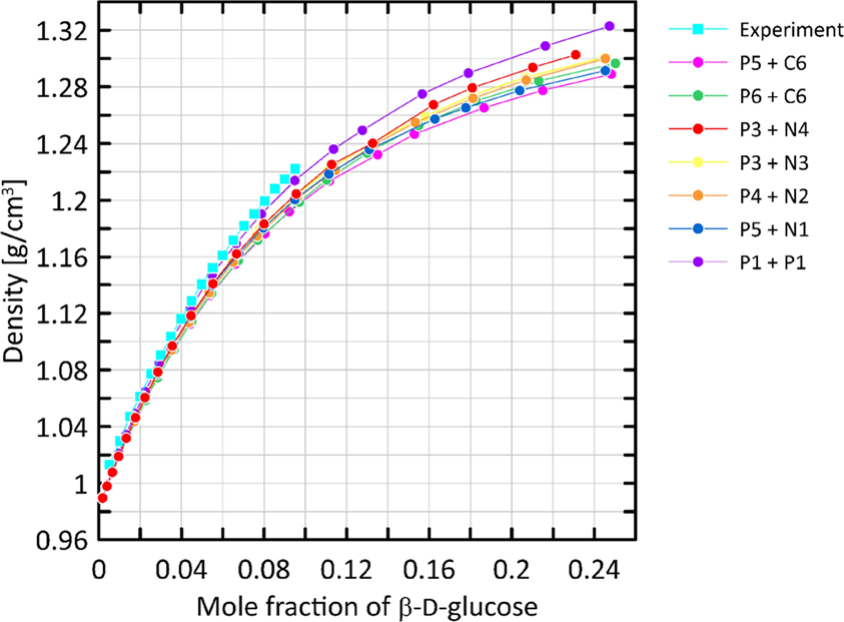
Density of aqueous solutions of β-d-glucopyranose as a function of the molar ratio. Results from CG simulations (circles) are compared with the experimental data^54^ (squares).

Our CG model was tested against its capability to reproduce the density of aqueous solutions of glucose. A systematic set of CG simulations was carried out at different glucose concentrations and compared with the experimental data. Figure 7 shows the graphical illustration of such comparison. The experimental densities are reproduced with a satisfactory accuracy up to maximal experimentally measured concentrations of ca. 0.1 molar fraction. The final P3 + N4 combination of bead types is associated with the error of prediction of ca. 1.5% and is the second best among all tested nonbonded parameters. However, as previously mentioned, all investigated combinations offer a similar level of agreement with reference data. Moreover, we did not observe any spurious sugar-sugar aggregation at high concentrations, up to 0.25 molar ratio.

### 5.4 Self-Assembly Properties

Because of a large polarity of carbohydrate molecules, the self-aggregation properties can often be seen as spurious artifact being a result of inaccuracies in the force field parameters.^27,32^ However, there exist a series of supramolecular structures created by self-interacting carbohydrate oligo- and polymers. The examples include helical structures created by curdlan chains,^55,56^ double amylose helix,^57^ and amylose V-helix present in some nonaqueous solutions^58^ or cellulose aggregation resulting in cellulose II-like complexes.^59^ The capabilities of the newly developed model were tested in the context of either spontaneous creating the carbohydrate aggregates or maintaining the structures initiated from the molecular geometries roughly corresponding to the given complex.

It was found that the double-stranded amylose helix of the structural features based on the XRD data is unstable and undergoes unfolding within several nanoseconds. This observation relies on the unbiased MD simulation in aqueous solution. The capability of the model to maintain the amylose V-helix in nonaqueous solutions was investigated in a systematic manner by dissolving the V-helix-like structure in a series of artificial solvents, composed of uniform beads of the following types: C1, C2, C3, C4, C5, and C6. However, in none of the cases, the initial structure was maintained. Instead, more compact aggregates have been formed in the presence of less polar solvents (beads C1 and C2), whereas unfolding of the V-helix occurred in polar solvents (beads C3 to C6).

**Figure 8:**
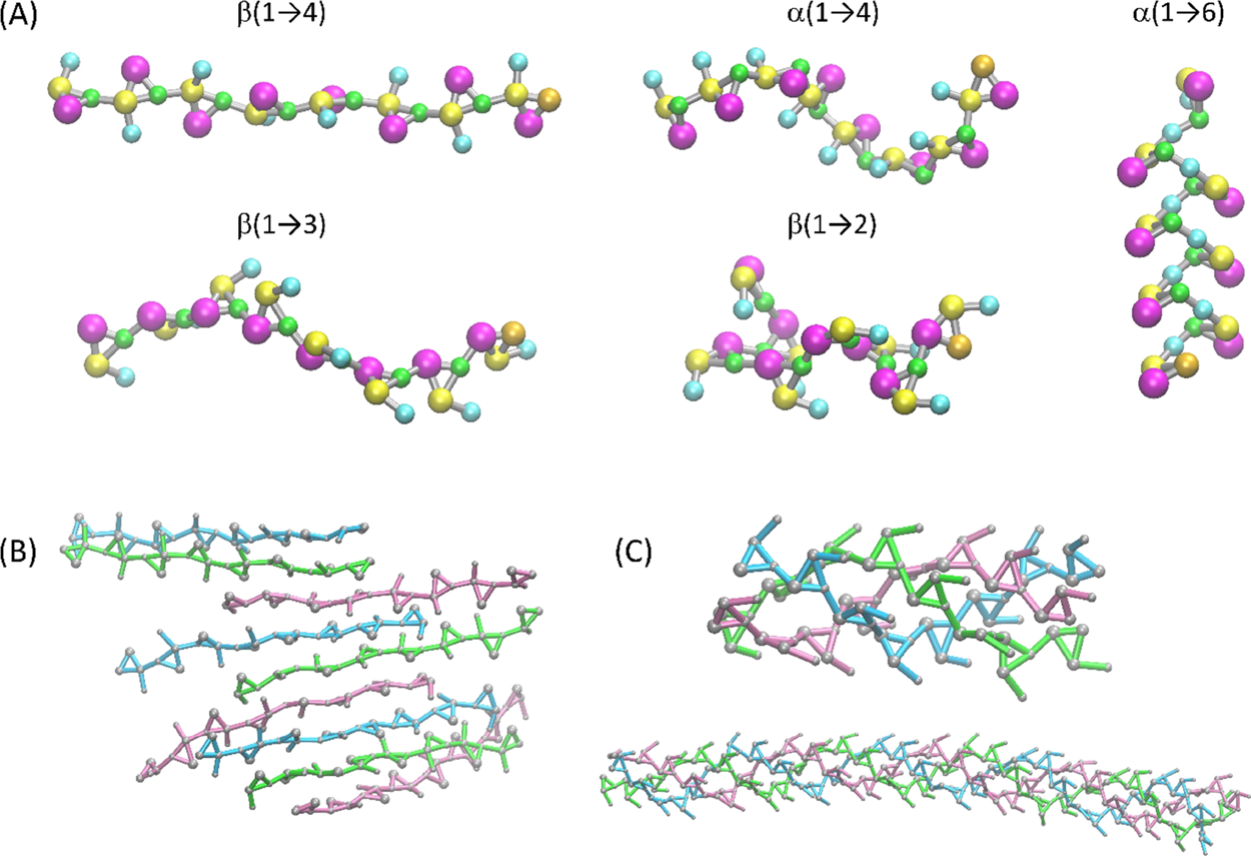
(A) Exemplary snapshots from the MD simulation of octamers of glucopyranose, exploiting different types of glycosidic linkages. (B) Cellulose II-like sheet formed during the simulation of aqueous solution of cellulose oligomers. (C) Triple helices of curdlan formed during the simulation of the aqueous solution of curdlan oligomers (*up*) or obtained as a result of simulation of the XRD data-based structure (*down*).

On the other hand, the unbiased MD simulations of concentrated solutions of octamers with the β(1 → 4) (cellulose) and β(1 → 3) (curdlan) linkages resulted in spontaneous formation of structures corresponding to curdlan triple helices and cellulose II-like sheets. In the latter case, the sheets exhibited a tendency to further aggregation, creating clusters that resemble the structure of the cellulose II crystal. The exemplary snapshots from MD simulations are shown in Figure 8. Additionally, we have studied the structural parameters of the curdlan triple helix composed of longer 32-residue-long chains. The helix length (per 1 helix turn) predicted by CG simulation is equal to ca. 1.95 nm, which is in between the predictions of the GROMOS 56A6_CARBO_R_ united-atom force field,^60^ that is, 1.79 nm for a single helix in solution and the value of 2.02 nm for helices packed in parallel in the supramolecular sheet.^61^

At the stage of testing various nonbonded parameters, we have observed that exchanging the R bead type (one of the beads defining the glucose ring) into the S one resulted in alterations of the aggregation properties. Namely, the carbohydrate–carbohydrate interactions become less attractive and, as a consequence, the events of self-aggregation less frequent. Still, at sufficiently high concentrations, aggregates of the types described above were possible to observe.

Thus, concluding, our CG model is capable of predicting the carbohydrate self-assembly properties in the cases where aggregation is driven by the contact of aliphatic patches on the pyranose rings and, possibly, supported by hydrogen interactions. Such a situation occurs primarily in the case of chains containing equatorial-equatorial glycosidic linkages. On the other hand, the structures supported mainly by hydrogen bonding are poorly reflected by the model, probably due to the directional character of such interactions, which is hardly reproduced by the LJ interactions between particular beads.

### 5.5 Interactions with the Lipid Bilayer

**Figure 9:**
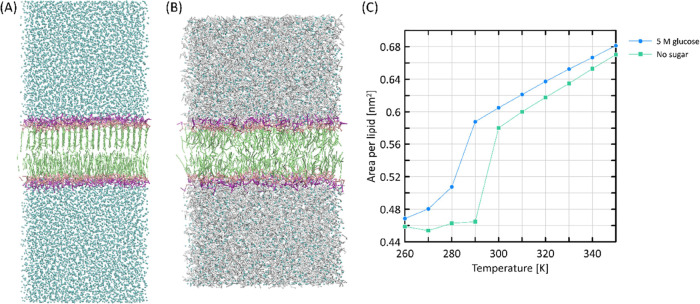
(A, B) Snapshots of CG DPPC bilayers at *T* = 290 K: (A) gel phase formed in pure water; (B) fluid phase stabilized in the presence of 4 M glucose. Lipid tails are depicted in green, head groups are shown in pink and violet, and glucose molecules shown in white. Water beads are represented by blue balls. (C) Average area per lipid of a DPPC bilayer as a function of temperature. Glucose-free (green) and 5 M glucose solution (blue) systems were considered.

Carbohydrates are capable of acting as cryo- and anhydro-protective agents for lipid membranes.^62^ One of the possible mechanisms of this effect relies on alterations of the main phase transition temperature of the lipid bilayer, which is lowered in the presence of carbohydrates. This phenomenon has been studied by means of both AA^63−65^ and CG^23^ simulations. Our model was tested against the capabilities to reproduce this effect. Several CG simulations of the DPPC lipid bilayer were carried out either in the absence or the presence of ca. 5 M glucose and with the temperature varying in the range of 260–350 K. The effect of carbohydrate influence was monitored by the area per lipid parameter. Figure 9 illustrates the results.

There exists a striking difference between the behavior of the two systems: the pure DPPC system adopts a sharp increase of area per lipid (APL) from ca. 0.46 to ca. 0.58 nm^2^ around 295 K, characteristic of a transition from a gel phase to a fluid phase. The glucose-containing system exhibits similar phase transition but at lower temperature, equal to ca. 283 K. At even lower temperatures (260–280 K), the glucose-containing system displays systematically higher APL values, whereas at the elevated temperatures (300–350 K), the thermal expansivity (i.e., the slope of the APL vs temperature curves) is slightly smaller in the presence of the sugars. All these findings confirm that glucose influences the structure of the lipid bilayer, increasing the stability of the fluid phase. The insight into MD trajectories clearly shows that the lower APL values are correlated with the conformation of the lipid tails forming the inner part of the bilayer (Figure 9). In the pure system, the DPPC tails below 290 K adopted am extended, straight structure, whereas in the presence of glucose, they remain more disordered.

In analogy to the results reported for Martini 2,^23^ also in this case, the analysis of MD trajectories indicates that water-replacement hypothesis^64,65^ can be evoked to explain the mechanism of the observed results. The carbohydrate molecules are able to bind to the lipid/water interface (via interaction with the lipid head groups) and can penetrate the membrane up to the level of the carbonyl groups. By the competition effect, the amount of water in the water/membrane interface is reduced. Although the physical mechanism of interactions between lipid bilayers, monosaccharides, and water is more complex and dependent on additional factors (e.g., sugar concentration),^66^ these findings demonstrate that the current model can reflect the carbohydrate-induced alteration in the structure of lipid bilayers.

### 5.6 Interactions with Proteins

Interactions of carbohydrates with proteins are essential for numerous processes occurring in living organisms. Thus, the protein–carbohydrate interactions are extensively studied by MD simulations at either the AA or CG level or resolution. In addition to the routine procedure relying on initiating the simulation with the correct, experimentally determined position of the ligand in the protein binding cavity, the extensive MD simulations may sporadically be used to blindly identify binding sites. The recent version of Martini proved to be capable of successfully carrying out such predictions.^67^ Presently, we have tested if the carbohydrate-binding cavities in several different proteins can be identified by using the Martini 3.0 force field in combination with our model. Carbohydrate–protein binding may, in principle, be problematic for classical force fields due to the fact that in the case of nonfunctionalized carbohydrates, binding is driven by the CH-π interactions.^68,69^ In spite of the lack of explicit functional form capable of mimicking this type of interactions, most of modern atomistic, biomolecular force fields offer reasonable predictions of the binding free energy, but some divergences arise when considering the quantitative descriptors of the binding pattern and strength.^70^ Additionally, either functionalized or unfunctionalized carbohydrates are highly hydrophilic, and binding to protein is associated with small or moderate changes in free energy. Thus, even small inaccuracies in the force field can result in its inapplicability to correctly predict any carbohydrate binding-related properties.

At this stage of the study, we investigated carbohydrate–protein binding in the three distinct systems: (1) endoglucanase I interacting with cellobiose (PDB: 2OVW); (2) maltose kinase interacting with maltose (PDB: 4O7P); (3) xylanase 10A interacting with glucose (PDB: 1I8A). Our aim was to check if the few tens microseconds-long MD simulation will allow to identify the experimentally determined carbohydrate binding sites.

**Figure 10:**
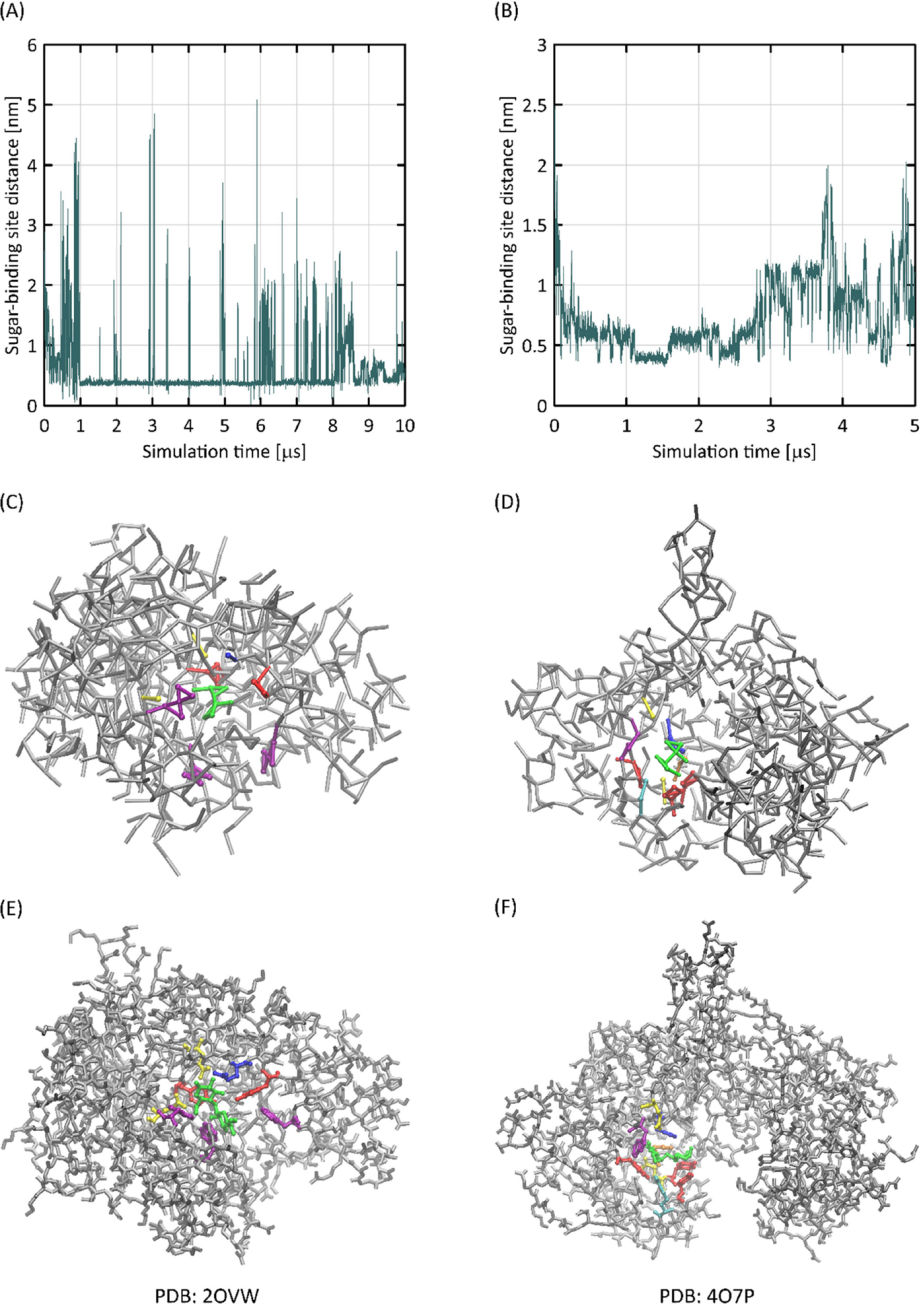
(A, B) Binding cavity-carbohydrate molecule distance recorded during MD simulations for protein–carbohydrate systems. Lowest values correspond to the binding event. (C, D) CG structures of the protein–carbohydrate complex and (E, F) atomistic, XRD structures, corresponding to them. In the case of panels (C–F), the same color code was used, highlighting, for example, carbohydrates (green) and two types of aromatic amino-acid residues, responsible for CH-π binding: tryptophane (violet) and tyrosine (red).

The results for systems (1) and (2) are illustrated in Figure 10. It appears that in both cases, the binding site is correctly identified with neither a priori assumed configuration nor any bias imposed to the system. The binding pattern is driven by interactions of “mixed” nature, that is, the CH-π interactions with tyrosines and tryptophans and hydrogen bonding with, for example, arginine or aspartic acid. Thus, even in spite of highly hydrophilic nature of unfunctionalized glucose di- and oligomers, the newly developed model is capable of correctly predicting the binding pocket, in analogy to noncarbohydrate ligands and their protein molecular targets, as described in a previous study.^67^

Interestingly, the same set of parameters applied for the system in which carbohydrate binding is driven mainly by the CH-π interactions with only a minor (or none) contribution of polar interactions gives opposite results. Such an example is xylanase 10A (structure deposited at PDB:1I8A), where the bound ligand is β-glucopyranose and the binding pocket is created by the two tryptophan sidechains. In this case, we did not observe any specific binding with the contribution of the experimentally identified binding pocket. Some binding events were observed when artificially reducing the hydrophilicity of the glucose beads. However, as such reduction is significant (e.g., converting N4 bead types into C4 and P3 into P2), this type of alteration is not recommended, as it will likely lead to numerous artifacts, associated with the presence of “hydrophobic sugars” in the system.

In conclusions, we state that our CG model is capable of identifying the carbohydrate binding pockets in the “blind” MD simulations, but the necessary condition for that is a large contribution of polar interactions that provide the driving force for binding. Otherwise, when binding is driven solely by the CH-π interactions, such predictions may not be successful. On the other hand, quantitative prediction of the energy involved in the CH-π interactions in carbohydrate–protein complexes can be problematic even in the case of atomistic force fields.^70^

## 6 Model Limitations and Potential Refinements/Extensions

The developed CG force field contains a series of limitations which, as a rule, are a consequence of coarse-grained resolution of the underlying model.

1. The interactions with a strong orientation dependence (e.g. hydrogen bonding) are modeled by orientation-free LJ interactions between beads. As a consequence, hydrogen bonding-mediated self-assembly in some carbohydrate-containing systems may not be accurately reproduced. This concerns mainly the formation of double-helix of amylose in polar solvents and amylose V-helix in non- or weakly polar solvents. On the other hand, self-assembly of “hybrid” nature, induced by both polar- and non-polar contacts (e.g., triple helices of curdlan and cellulose II structure) are reproduced correctly (Section 5.3),
2. Binding of small carbohydrate molecules by proteins driven nearly exclusively by CH-π interactions is not correctly reflected. The reason is the lack of sufficiently hydrophobic beads representing the nonpolar patch of the glucose ring, responsible for CH-π binding. On the contrary, carbohydrate–protein binding occurring with larger contribution of polar interactions is reflected properly (Section 5.4).
3. Some of the conformational rearrangements occurring within the carbohydrate backbone are correlated with the alterations of the geometry of pseudo-chiral centers, described by the “improper-dihedral” force field terms. These terms are represented by parabolic potentials with single minimum; thus, they are not capable of reflecting any nonharmonic conformational changes. This issue concerns mostly the β(1 → 4) linkages. As a consequence, the long polysaccharide chains containing this linkage type (cellulose) will suffer from the lack of inherent conformational “kinks”, which will result in apparent stiffening of the chain and influence several polymer properties (e.g., persistence length, end-to-end distance). However, in the studied oligomeric systems, this effect is likely to have a limited influence.
4. The conformational transitions of the chair-to-inverted chair or chair-to-boat type occurring within pyranose ring are not modeled at all. This is partially due to the complexity of the problem considered in the context of limitations of the functional forms of the bonded potential combined with the simplicity of the accepted CG representation. Nevertheless, this issue does not seem to be especially important for currently considered glucopyranose-based saccharides because of the fact that the glucopyranose ring belongs to the most rigid ones and the population of non-^4^C_1_ conformers is usually negligible under standard conditions.
5. In connection to points 3 and 4, it is worth mentioning that the atomistic force field differ significantly in their predictions related to free energy levels associated with some secondary or tertiary conformers. This includes, for example, the nonstandard pyranose ring conformers^71^ and rotamers of glycosidic bonds (Lutsyk and Plazinski, unpublished data). As CG models rely usually on the AA reference data, some of these uncertainties may also be treated as a weak point of the current CG model.
6. The developed set of parameters was tested only in the context of various homopolymers. Although the currently proposed set is theoretically capable to simulate unbranched heteropolymers as well as branched polysaccharides (under assumption that the branching type corresponds to any of the glycosidic linkages considered in this work), systems of those character were not studied so far. Therefore, it is expected that some refinements may be necessary, at least at the level of bond-length alterations resulting from minor redefinitions of beads.

The issues mentioned in points 1 and 2 may potentially be addressed by introducing alternative sets of parameters designed with the aim to deal with those problematic situations. For instance, a separate set of parameters can be introduced to simulate amylose in nonpolar solvents (in analogy to the previous version of Martini), whereas CH-π binding can be enforced by intentionally decreasing the polarity of certain beads in the carbohydrate model (ignoring the potential consequences for, for example, log *P* value). Furthermore, maintaining the supramolecular structures of long carbohydrate chains may be supported by introducing rationally designed constraints between particular fragments of the system (in analogy to the elastic network approach routinely applied in the protein-dedicated editions of Martini). In order to capture the rare conformational transitions (points 3 and 4), nonstandard functional forms of some bonded potential may be necessary. This includes, for example, GROMACS-compatible tabulated potentials. Some of these solutions will be explored in our future studies.

## 7 Conclusions

The CG model, compatible with the new version (3.0) of the Martini biomolecular force field and concerning glucopyranose-based saccharides has been developed. The proposed parameters that constitute the model are described and discussed in the previous sections of the article, whereas the Supporting Information contains the program facilitating the automated generation of the force field files (topologies and structures in the GROMACS format) for carbohydrate chains of user-defined lengths. The model covers the parameters for glucose-containing saccharides, in particular, glucopyranose monomers (separately for α or β anomers), disaccharides containing the linkages of the β(1 → 2), β(1 → 3), α(1 → 4), β(1 → 4), or α(1 → 6) types, as well as oligo- and polysaccharides exploiting such linkages. The capabilities of our model were tested through multiple MD simulations, confirming that the properties of glucose monomers and polymers simulated at the CG level are in accordance with the predictions of AA models and with the available experimental data. More precisely, we were able to satisfactorily reproduce the density profiles of glucose solutions, experimental log *P* values, and a series of conformational properties of homo-oligomeric chains. The compatibility of our newly proposed CG model with the Martini 3.0 CG force field arises from adopting a similar parameterization strategy and using the bead definition introduced for Martini 3.0. In this context, we also tested our model in the system where carbohydrates can interact with other biomolecules, such as lipid bilayers and proteins. In the context of sugar–lipid bilayer interactions, our model correctly predicts the stabilizing effect of the concentrated solution of glucose on the bilayer structure, protecting it from transition to gel phase. In the case of interactions with proteins, the model is capable of correctly identifying the binding pocket of carbohydrate-binding proteins when the binding occurs via both polar and CH-π interactions. However, it seems that the model has limited capabilities to identify the binding pockets in system where carbohydrate binding is driven primarily through CH-π interactions. The final model may serve as a convenient starting point to further extensions, involving either glucopyranose-based functionalized saccharides or other group of saccharides exploiting different residues and glycosidic linkage types. Developing and validating such extensions will be the subject of our future investigations.

## Supporting Information Available

Python script (*carbo2martini3.py*) allowing to generate primitive starting geometries and force field files (in the GROMACS format) for various homopolymers of glucopyranose (ZIP)
